# Canine and Feline Epididymal Semen—A Plentiful Source of Gametes

**DOI:** 10.3390/ani11102961

**Published:** 2021-10-14

**Authors:** Hiba Ali Hassan, Guillaume Domain, Gaia Cecilia Luvoni, Rana Chaaya, Ann Van Soom, Eline Wydooghe

**Affiliations:** 1 Department of Reproduction, Obstetrics and Herd Health, Faculty of Veterinary Medicine, Ghent University, Salisburylaan 133, 9820 Merelbeke, Belgium; Guillaume.Domain@ugent.be (G.D.); ann.vansoom@ugent.be (A.V.S.); eline.wydooghe@ugent.be (E.W.); 2 Faculty of Agronomy and Veterinary Medicine, Lebanese University, 2703 Beirut, Dekwaneh, Lebanon; chaaya_rana@hotmail.com; 3 Dipartimento di Scienze Veterinarie per la Salute, la Produzione Animale e la Sicurezza Alimentare, Università degli Studi di Milano, Via dell’Università 6, 26900 Lodi, Italy; cecilia.luvoni@unimi.it

**Keywords:** epididymis, spermatozoa, epididymal semen, maturation, collection

## Abstract

**Simple Summary:**

The epididymis is a source of fertile spermatozoa. For some males, preserving spermatozoa that are stored in the epididymis might be an ultimate attempt for gamete preservation. The quality of epididymal semen is different from ejaculated semen in various animal species. Although assisted reproductive technologies (ART) have been introduced in cats as a tool to preserve valuable genetics of endangered wild felids, epididymal semen cryopreservation is still suboptimal in dogs. Therefore, in this paper, we carried out a review to list the morphological changes of spermatozoa during epididymal transit alongside with the potential that holds in the epididymal semen in dogs and cats. We believe that better comprehension of epididymal semen collection method, quality and freezability may aid in optimizing cryopreservation and enhance different applications of ART.

**Abstract:**

Canine and feline epididymal semen provide an additional source of gametes to preserve the genetics of valuable breeding dogs and tomcats, especially for those that fail to ejaculate, need castration as a therapy or die unexpectedly. Moreover, since it is quite common to perform castration of non-breeding dogs and cats, the development of a gene bank of epididymal semen collected after castration would greatly contribute to increase the genetic diversity in dogs and cats. Collection and cryopreservation of epididymal semen necessitates a full understanding of the function of the epididymis and of the characteristics of epididymal spermatozoa as opposed to ejaculated semen. During collection of epididymal semen, specific factors may have a negative effect on epididymal semen quality and freezability. Accordingly, the elimination of these triggers could enhance epididymal semen freezability and consequently positively influence post-thaw semen quality and outcome for different ARTs.

## 1. Introduction

Epididymal spermatozoa remain a kind of enigma. They are often described as immature, immotile, and infertile sperm cells, yet the epididymis is the organ where spermatozoa mature, acquire motility, and become fully fertile. This coiled duct constitutes a reservoir for millions of spermatozoa before ejaculation; therefore, the epididymis is a valuable source for spermatozoa in stud dogs or endangered canids and felids that die suddenly or need to be castrated. Semen donation and banking in pedigree dogs has become more and more important and can serve as a central tool to prevent inbreeding in certain dog breeds [1]. Moreover, in cats, a species notoriously difficult to collect semen from, epididymal sperm may serve as an extra and easily accessible source of gametes [2]. However, epididymal spermatozoa are different from ejaculated spermatozoa in certain morphological and functional characteristics. They differ in their maturation stages according to the part of the epididymis from which they have been collected. In other words, a sperm cell found in the caput of the epididymis possesses characteristics that are different from sperm cells found in the corpus or cauda of the same epididymis, such as the cytoplasmic droplet position. The droplet is located in the proximal region of the midpiece in a caput-spermatozoon and in the distal region of the midpiece in a corpus-spermatozoon [3]. As spermatozoa pass through the epididymis, they undergo a series of maturational changes, dependent on the different functions of each part of the epididymis. The functional dialogue between epididymal cells and spermatozoa, and subsequent modifications to the spermatozoa, mainly take place in the caput and corpus of the epididymis, whereas the main function of the cauda is to store spermatozoa [4]. A better understanding of the function of this organ and of the chronology of sperm maturation may elucidate the etiological background of many idiopathic subfertilities that are related to disorders in sperm maturation. Therefore, the aim of this review is to highlight the characteristics of epididymal semen in dogs and cats and to identify critical factors that may be responsible for specific morpho-functional characteristics of epididymal sperm cells and their possible clinical and laboratory relevance in subfertility diagnosis and treatment, as well in semen preservation procedures.

## 2. Morphological Changes in the Spermatozoa during Epididymal Transit

**Figure 1:**
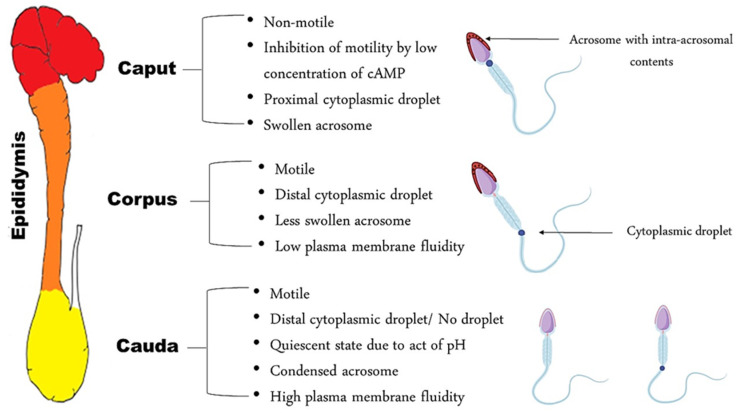
Schematic representation of spermatozoa characteristics in each epididymal segment.

At the exit of the testis, spermatozoa are non-fertile, despite the fact that they are morphologically differentiated. In the initial segment of the epididymis, the sperm cell is immotile—unable to fertilize an oocyte and has a proximal cytoplasmic droplet—close to the neck [3]. As the spermatozoa move along the epididymis, changes in plasma membrane fluidity take place, as well as the acquisition of motility [5,6]. Morphologically, the most obvious change is the migration of the cytoplasmic droplet along the mid-piece to a distal position, resulting finally in detachment of the droplet; this occurs in the corpus, region 3, of the epididymis in dogs and at the transition between the caput and corpus in cats, region 4 [7]. In the cauda, the spermatozoa become motile and acquire a fertilization ability, while some of them still have a distal cytoplasmic droplet, near the annulus (Figure 1).

There is a presupposition that sperm head morphology can reflect the status of sperm DNA since the latter occupies almost 90% of the spermatozoal head [8]. This was proved in bulls after using the Fourier harmonic analysis as an approach to correlate spermatozoa morphometry with sperm chromatin integrity [9]. Notably, in dogs, a significant relationship between sperm morphometry and chromatin integrity was present [8] in contrast to cats, where the sperm DNA fragmentation index was found to be independent from sperm head morphometry [10]. This may indicate that different factors other than chromatin compaction might affect the sperm head shape, especially since the sperm head is not solely composed of DNA. The acrosome, which plays a key role in fertilization, is located on the anterior part of the sperm nucleus. An important change necessary for capacitation is the condensation and contraction of the acrosomal cap during epididymal migration of the spermatozoon [3]. This reshaping is reflected by the enlarged acrosomal dimensions of spermatozoa present in the caput when compared to those in the cauda [11]; this has also been confirmed in dogs through the presence of a higher number of canine spermatozoa with swollen acrosomes in the caput and in the corpus, as compared to the cauda [3]. Concomitant to acrosomal reshaping, the acquisition of motility and reorganization of the composition of the plasma membrane occurs. Regarding motility, caput spermatozoa are nearly immotile in dogs [12] and cats [13]; they acquire motility gradually as they migrate caudally within the epididymis. Therefore, since AI and IVF require motile spermatozoa, sperm from both the corpus and cauda can be used. Caput spermatozoa can be extracted but are immotile and only useful for ICSI, especially in cats [13].

Several factors are considered to have an effect on sperm motility. The composition of the sperm plasma membrane is important for sperm motility; in dogs, the relative abundance of fatty acids increases gradually in the sperm plasma membrane as the sperm migrates into the cauda and acquires motility [6].

The unique features of the epididymal journey of the spermatozoa differ between species but have not yet been investigated in detail for several mammals. It is known that, in order to achieve fertilization competence, sperm cell modifications have to take place, such as cytoplasmic droplet migration, acrosomal cap condensation, changes in the composition of membranes, motility development, and the acquisition of fertility [5]. Several features of epididymal sperm maturation, which are either different (Table 1) or common, (Table 2) between dogs and cats are of utmost importance for developing precise protocols for semen evaluation features in epididymal sperm maturation, evaluation, and freezing, as well as for developing species-specific sperm fertility markers.

## 3. Anatomical and Functional Characteristics of the Epididymis

**Figure 2:**
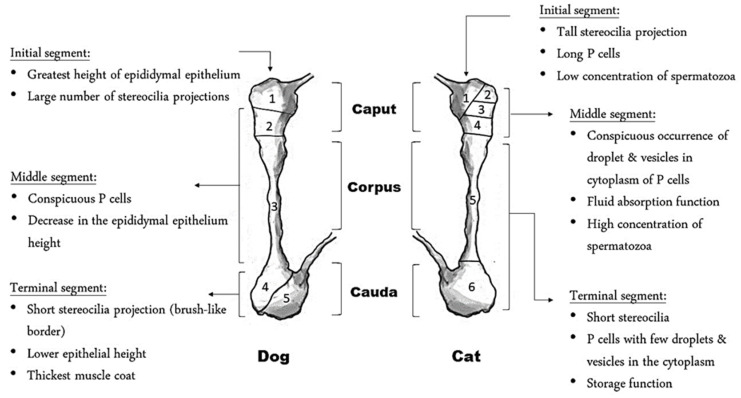
Histological difference between the epididymis of the dog and cat. P refers to principal cells.

The epididymis is a long, coiled tube that is attached along the dorsolateral surface of the testis in dogs and across the craniolateral aspect of the testis in cats [21]. The epididymal transit of spermatozoa takes about 14 days in dogs [22], whereas the exact time of transit is not yet known in cats. During this transit, the epididymis provides an optimal microenvironment for sperm maturation and storage in a viable state ready for ejaculation. This role is well established by the production of epididymal plasma, which differs in composition within epididymal segments, and which plays an important role in the maturation of sperm cells [23]. Epididymal proteins interact directly and/or indirectly with the spermatozoa, thus altering their function and morphology. Some proteins bind to the surface of the sperm cell, whereas other proteins remain within the lumen of the epididymis for only a short time [23]. It is thought that these short-acting proteins block certain receptors present on the surface of sperm cells that are activated during fertilization [4]. The storage time is dependent on the frequency of ejaculation. Multiple ejaculations over a short period of time will result in a low semen volume. This may be the result of a limited storage capacity for semen and/or the level of secretion in the male accessory glands [24]. The epididymis forms a structure that can be divided into segments based on either function or location. Macroscopically, the terms used are head, body, and tail of the epididymis that correspond to caput, corpus, and cauda, respectively. Initial, middle, and terminal segments are used as wording when the epididymis is subdivided based on functional differences [25]. Interestingly, both the initial and middle segments are located in the caput of the feline epididymis, while in dogs, the initial and middle segments are located in the caput and corpus of the epididymis, respectively (Figure 2). The precise number of epididymal regions is species-specific. The feline epididymis can be divided into six distinctive histological regions, whereas the epididymis of the dog is subdivided into five regions [9,10]. These regions show differentiating features with respect to epithelial height, length of stereocilia, luminal diameter, and thickness of the muscular wall [9].

The regionalized functionality of this long tubule is related to the different structural regions of the epididymal duct-epithelium lining. Along the entire length of the duct, principal cells are the most numerous in the epididymal epithelium; however, they show different characteristics according to different segments [7]. They are characterized by stereocilia, which are branched microvilli, increasing the surface area and hence contributing to the absorption of luminal fluid. These stereocilia are most prominent in the initial segment of the epididymis, reflecting the main function of this segment, which is the absorption of testicular fluid [23]. In addition, the presence of an abundant amount of lysosomes and of the large Golgi apparatus in the principal cells, especially in the proximal parts of the epididymis, contributes to the parallel function of the epididymis in these regions in digestion and secretion of materials [26]. Such materials are vesicle-like structures called epididymosomes. They are released from the epididymal epithelium by an apocrine secretion mechanism, consisting of a diameter ranging between 50 and 500 nm [27]. The role of epididymosomes is to transport proteins that lack certain signaling peptides from the epididymal lumen to the binding site on the spermatozoa [28]. Similar to spermatozoa, epididymosomes within different regions in the epididymis exhibit different epididymal content [29]. This reflects the presence of mixed spermatozoa in various stages of maturation with different macromolecular demands. Epididymosomes were found to control sperm motility, exert a protective effect against oxidative stress, eliminate defective sperm cells, and induce the spermatozoa’s ability to interact with the zona pellucida [30]. These functions originate from the protein(s) associated with epididymosomes. For example, glutathione peroxidase (GPX5) was found to be associated with rat epididymosomes. GPX5 has an antioxidant effect by catalyzing the reduction in hydrogen peroxide and lipid peroxides, hence preserving spermatozoa DNA integrity and the plasma membrane [30]. Evidently, in cats, upon co-incubation of testicular spermatozoa with epididymosomes, a higher motility was obtained with a significant increase in the percentage of spermatozoa expressing cenexin—a non-signal peptide protein that is transported by epididymosomes—when compared to the control group [28]. This suggests that epididymosomes aid in the maturation process of epididymal spermatozoa by suppling proteins that protect the cells during transit and aid in motility acquisition. Consequently, more studies are needed to detect the protective effect of epididymosome upon its addition to semen samples prior to cryopreservation. In association with epididymosomes, the ionic composition of the epididymal fluid plays a crucial role in the regulation of luminal acidification and sperm functionality [31]. The former is achieved through the secretion of H^+^ by proton pumping ATPase in response to the activation of bicarbonate-sensitive adenylyl cyclase after HCO_3_^−^- and Cl^−^ secretion by the principal cells, whereas sperm functionality, mainly motility, is altered by the inflow of Ca^2+^ into the spermatozoa through cation channels (CATSPER) located in the principal piece of the sperm flagellum [31].

## 4. Collection of Epididymal Semen in Cats and Dogs

The interest in semen cryopreservation in dogs and cats is rapidly growing. Research in different species focused on the optimization of cryopreservation procedures to improve semen quality has created great advances in the field during the last decade. Many factors are known to affect semen quality during cryopreservation. For instance, the method of semen collection has an important impact on semen quality, thus being the first bead in the rosary [32]. Semen collection methods can be categorized into two groups: in vivo collection (mainly ejaculated semen) and in vitro collection (testicular or mainly epididymal semen). Various methods are employed for epididymal semen collection, such as epididymal mincing, in vitro epididymal sperm aspiration post-epididymectomy, and in vivo percutaneous epididymal sperm aspiration (PESA). Epididymal mincing or the float-up method is an excellent, non-repeatable collection technique that can be performed after orchiectomy or post-mortem. The epididymis is dissected from the testicle and subjected to parallel incisions after being suspended in a medium (in most cases semen extender). The incubation of the epididymis for a few minutes will allow the sperm cells to be released into the medium [33]. Although blood vessels are avoided during epididymal incision, contamination with blood is frequently observed when this technique is applied [33]. This may preclude its suitability for semen cryopreservation. Concerning epididymal sperm aspiration, this collection method is performed by aspirating semen, using a needle connected to a syringe, directly into the epididymis (cauda). This technique can be performed either under direct visualization of the isolated epididymis (in vitro) or under general anesthesia of the dog (in vivo; PESA) [34]. PESA is mainly applied when orchiectomy cannot be performed due to medical reasons or because of the dog’s owner’s reasons. PESA can be used also as a diagnostic tool to assess azoospermia. For instance, human epididymal spermatozoa collected by PESA are used for intracytoplasmic sperm injection (ICSI) [35]; hence, PESA is a feasible, efficient, and repetitive procedure. However, there are some downsides associated with PESA. In rats [36], inflammatory and stereological alterations in the epididymides were detected after repeated puncture sessions. To our knowledge, investigations into possible side effects of PESA have not been conducted in cats, but Attia et al. (2000) found that epididymal aspiration induced a transient formation of anti-sperm IgG on spermatozoa, with no negative effects on total spermatozoa output or motility [37]. In addition to PESA, epididymal mincing is an excellent, non-repeatable collection technique that can be performed after orchiectomy or post-mortem. The quality of canine epididymal semen has been examined using three different (listed above) collection methods [34]. Although epididymal mincing required more time and precision to avoid vessel puncturing and consequent blood contamination, it yielded the highest semen volume concurrently with a high total sperm count and good motility when compared to PESA and in vitro sperm aspiration, respectively.

## 5. Evaluation of Epididymal Sperm Quality

Despite the on-going dilemma regarding the definition of fertility, i.e., whether it is the ability to conceive or to produce offspring, a legitimate question remains: is epididymal semen fertile? In order to ascertain epididymal semen fertility, many studies have been conducted to examine the different parameters that are thought to affect fertility, such as morphology [34], motility [38], sperm chromatin integrity [10,39], and acrosome and plasma membrane integrity [3]. Among these parameters, sperm DNA integrity is of the utmost importance and can be used as a potential marker for fertility, more so than conventional semen parameters [39]. An intact spermatozoon exhibiting normal motility and morphology can carry damaged DNA that will affect embryonic development and subsequent pregnancy rates [20,40]. Sperm chromatin integrity is assessed by the DNA fragmentation index (DFI), which refers to the percentage of spermatozoa with damaged DNA or abnormal protamination [41]. A semen sample is considered of low quality when the DFI percentage is higher than its threshold. This subfertility marker is species specific. For instance, the DFI threshold percentage is 30% in humans [42], 6% in pigs, and 28% in stallions [41]. Little is known about this topic in dogs and cats, and the DFI threshold has not yet been determined. The DFI of sperm cells in ejaculated semen of fertile dogs was significantly lower when compared to subfertile dogs with mean values of 5.9% and 13.2%, respectively [43]. In cats, the DFI ranged from 2.4 to 5.7% in fresh epididymal semen [20] versus 21–31% in frozen–thawed epididymal semen [44] when using the same method of detection. This discrepancy can be linked to either the large individual variation in semen quality in felids [20] or to the fact that cryopreservation can induce DNA fragmentation [44]. Almost 80% of idiopathic cases of infertility are related to low DNA integrity. Extrinsic factors, such as management and processing factors (centrifugation, method of collection and cryopreservation, and different types of extenders), can also exert a negative influence on DNA integrity [41]. For instance, chilled canine ejaculated semen displays slightly higher levels of DFI when incubated for 10 days in an egg yolk-based extender compared to an egg yolk-free extender [39], whereas intrinsic factors are more related to spermatozoa with defective chromatin integrity, ROS generation, and abortive apoptosis [41]. When comparing applications of ART in domestic cats and dogs, it is obvious that the cat comes first in this area of research. Due to problems with canine oocyte maturation in vitro, canine IVF is still in its infancy, whereas feline IVF can be routinely applied [45].

To the best of our knowledge, no epididymal spermatozoa have been used yet for in vitro fertilization in dogs. Epididymal semen (from cauda) has been used with good results in canine AI.

Frozen–thawed epididymal dog semen has given rise to the whelping of two puppies (conception rate 6.3%) and three puppies (conception rate 16.7%) through unilateral intrauterine insemination and intratubal insemination, respectively [46]. The conception rate increased up to 80% when post-thaw canine caudal epididymal sperm cells were sensitized by prostatic fluid prior to freezing [47]. Intravaginal insemination of fresh caudal epididymal semen resulted in eight live-born puppies in an American Staffordshire terrier [48] and of chilled epididymal semen collected post-mortem in the birth of one live Chihuahua pup [49].

In cats, many different methods of ART have been applied using epididymal feline semen (corpus and cauda) (Table 3). Advances in feline AI and IVF are considered important since the domestic cat serves as a perfect model for more than 36 species of wild felids that are classified as endangered; this will enable scientists to use similar techniques in wild felids [50].

## 6. Freezability of Epididymal Semen

The main objective in working with epididymal semen is to preserve the genetics of valuable males that have died unexpectedly. Hence, epididymal semen cryopreservation is a handy alternative when an estrous female is not available. Nonetheless, cryopreservation exerts a dramatic and negative effect on sperm viability and function [30]. When preserving semen, numerous factors may affect semen freezability and post-thaw quality, such as blood contamination, cytoplasmic droplet retention, and reactive oxygen species (ROS) [54,55]. Especially when the mincing technique is applied, epididymal semen is contaminated with RBCs that can be observed macroscopically most of the time. In the canine model, the addition of blood, up to 10% (*v*/*v*), in ejaculated semen did not have a negative impact on spermatozoa that were stored for 4 days at 4 °C, as opposed to spermatozoa that were undergoing cryopreservation and were negatively affected [56]. This could be related to the fact that RBC contains hemoglobin, which is released upon hemolysis triggered by a freezing–thawing process. Hemoglobin, a major source of toxic iron, amplifies the production of ROS [56]. This increase in ROS results in oxidative stress due to the imbalance between ROS and antioxidants. The oxidative stress mechanism targets mainly the sperm plasma membrane, affecting its fluidity and integrity as result of the lipid peroxidation phenomenon [57]. This could also be a problem in freezing sperm cells with a retained cytoplasmic droplet. The retention of a cytoplasmic droplet stimulates the production of ROS by released enzymes, such as glucose 6-phosphate dehydrogenase, found in excessive amounts within the cytoplasmic droplet. This enzyme fuels the generation of nicotinamide adenine dinucleotide phosphate (NADPH) that, in turn, stimulates the production of ROS [54,57]. Hence, the use of appropriate strategies to eliminate such detrimental factors and minimize the production of ROS is crucial, especially when epididymal semen samples are cryopreserved. Several protocols have been implemented to optimize semen sampling, especially by removing RBC prior to freezing. Such protocols may be based on the application of density gradient centrifugation, erythrocyte–sperm separation media, and microfluidic systems [58,59,60]. The efficacy of gradient centrifugation was confirmed by the successful separation of viable motile canine spermatozoa from dead immotile spermatozoa and RBC using four different types of density gradient centrifugation media [58]. Although the erythrocyte lysis buffer medium did not affect sperm morphology as well as acrosome and chromatin integrity of human sperm cells [59], it has not yet been used in canine or feline epididymal semen as a tool to remove RBC.

Semen freezability is also affected by the composition of the extender [61]. The extender must contain a balanced number of cryoprotective agents to protect spermatozoa from cold shocks and provide an exogenous source of energy. Additionally, cryoprotective agents must be low in concentration to avoid toxic shock [62]. Glycerol is the main cryoprotective agent used in freezing extenders in dogs and cats. While glycerol is only used in concentrations of up to 4% in cats because of the sensitivity of feline semen to its toxic effects, various regimens of glycerolization, ranging from 4% to 11% (*v*/*v*), exist in dogs [63,64]. In comparison to ejaculated semen, epididymal semen is less resilient to cryopreservation. Possible reasons could be the presence of the cytoplasmic droplet in epididymal semen and the absence of contact between epididymal spermatozoa and prostatic fluid. The sensitization of epididymal semen with prostatic fluid prior to freezing leads, however, to conflicting results, indicating the need for further research [65,66].

## 7. Conclusions

In conclusion, canine and feline epididymal semen are plentiful sources of gametes for developing a gene bank. Preserving genetic materials is a subject of interest, especially when endangered wild felids and canids or valuable breeding dogs or tomcats are involved. Optimizing the collection and cryopreservation of epididymal semen can be achieved by minimizing the action of detrimental factors that affect the cryopreservation of sperm. This can be one step forward in applying different ARTs in canine and field species.

## Figures and Tables

**Table 1:** Different features in epididymal sperm maturation in dogs and cats.

Different Features	Dog	Cat	References
Total sperm length	62.7 µm	58.6 µm	[14,15]
Acquisition of motility	In corpus	In caput-corpus junction	[7,16]
Motility mechanism	Supported by glycolysis	Supported by endogenous lipids	[7,17]
Migration of cytoplasmic droplet to end of midpiece	In corpus	In caput- corpus junction	[3,7]

**Table 2:** Common features of epididymal sperm maturation in dogs and cats.

Common Features	References
Fluid leaving the testis is reabsorbed in efferent duct and caput of the epididymis	[7,17]
Osmotic pressure within the epididymis increases caudally	[17,18]
Sperm plasma membrane is composed of large amount of phospholipids, cholesterol and glycoproteins	[6,19]
Cholesterol stiffens the sperm plasma membrane and increases its permeability	[6,7]
Glycoproteins coating the plasma membrane change in the same manner during epididymal transit	[19]
Percentage of spermatozoa with swollen acrosome and incomplete condensed chromatin decreases as sperm migrates distally along the epididymis	[3,15]
Presence of selective abnormal spermatozoa removal (phagocytosis)	[7,17]
Retained cytoplasmic droplets will be detached at or post-ejaculation	[20]
Spermatozoa contain only protamine P1 which renders chromatin relatively stable	[20]

**Table 3:** Application of ART in the domestic cat using frozen–thawed epididymal semen.

Method of ART	Region of Epididymal Sperm	Sperm Dose	Deposition Site	Conception Rate	Cleavage Rate	Reference
AI	cauda	5 × 10^7^	unilateral intrauterine	27.30%	-	[51]
AI	cauda	4 × 10^7^	unilateral intrauterine	28.60%	-	[19]
AI	cauda	1 × 10^7^	Intratubal	80%	-	[19]
			(24 hr post hCG injection)			
			Intratubal	20%	-	
			(30 hr post hCG injection)			
IVF	cauda	5 × 10^5^	-	-	33.33%	[52]
IVF	corpus	5 × 10^5^	-	-	32.03%	[52]
ICSI	cauda	single spermatozoa	-	-	82.20%	[53]
